# Supplementary material for: gcaPDA: a haplotype-resolved diploid assembler
Source: BMC Bioinformatics. 2022 Feb 14;23:68. doi: 10.1186/s12859-022-04591-4 (PMC8842951; doi:10.1186/s12859-022-04591-4)
Supplement: Supplementary file 1 — Additional file 1. Supplementary figures and tables. [file 12859_2022_4591_MOESM1_ESM.docx]

**Supplementary Figures**

Supplementary Figure 1. Comparison of framework of different diploid assemblers.

Supplementary Figure 2. The analysis workflow of gcaPDA.

Supplementary Figure 3. Genome survey of the SK x B73 F_1_ hybrid.

Supplementary Figure 4. Quality control of gamete cells based on SNP statistics.

Supplementary Figure 5. Distribution of distance between adjacent SNPs in the reconstructed haplotypes.

Supplementary Figure 6. Visualization of haplotype blocks of gamete cell S2.

Supplementary Figure 7. K-mer distribution of haplotype reads before and after normalization.

Supplementary Figure 8. Comparison of phasing accuracy of contigs of different assemblies for rice.

Supplementary Figure 9. Visualization of sequence alignments between gcaDPA assembly and parental genomes.

Supplementary Figure 10. Accumulated percentage of genomic k-mers covered in gamete cells reads.

**Supplementary Tables**

Supplementary Table 1. Statistics of simulated reads.

Supplementary Table 2. Statistics of sequencing reads of gamete cells.

Supplementary Table 3. Statistics of Hi-C sequencing reads.

Supplementary Table 4. Purge haplotigs from FALCON primary contigs.

Supplementary Table 5. Statistics of SNPs identified for gamete cells.

Supplementary Table 6. The statistic of HiFi reads of the MH63×ZS97 hybrid.

Supplementary Table 7. The statistic of single cell sequencing data of the tetrads of MH63×ZS97 hybrid.

Supplementary Table 8. The statistic of Hi-C reads of the MH63×ZS97 hybrid.

Supplementary Table 9. Evaluation of Hifiasm assembly, Trio assembly and gcaPDA assembly.

Supplementary Table 10. Statistics of haplotype blocks from the other haplotype.

Supplementary Table 11. Statistics of whole genome sequence comparison between haplotype assemblies and reference assemblies.

Supplementary Table 12. The computing resources used by differnet methods for rice F1.

Supplementary Table 13. The computing resources used by differnet methods for maize F1.


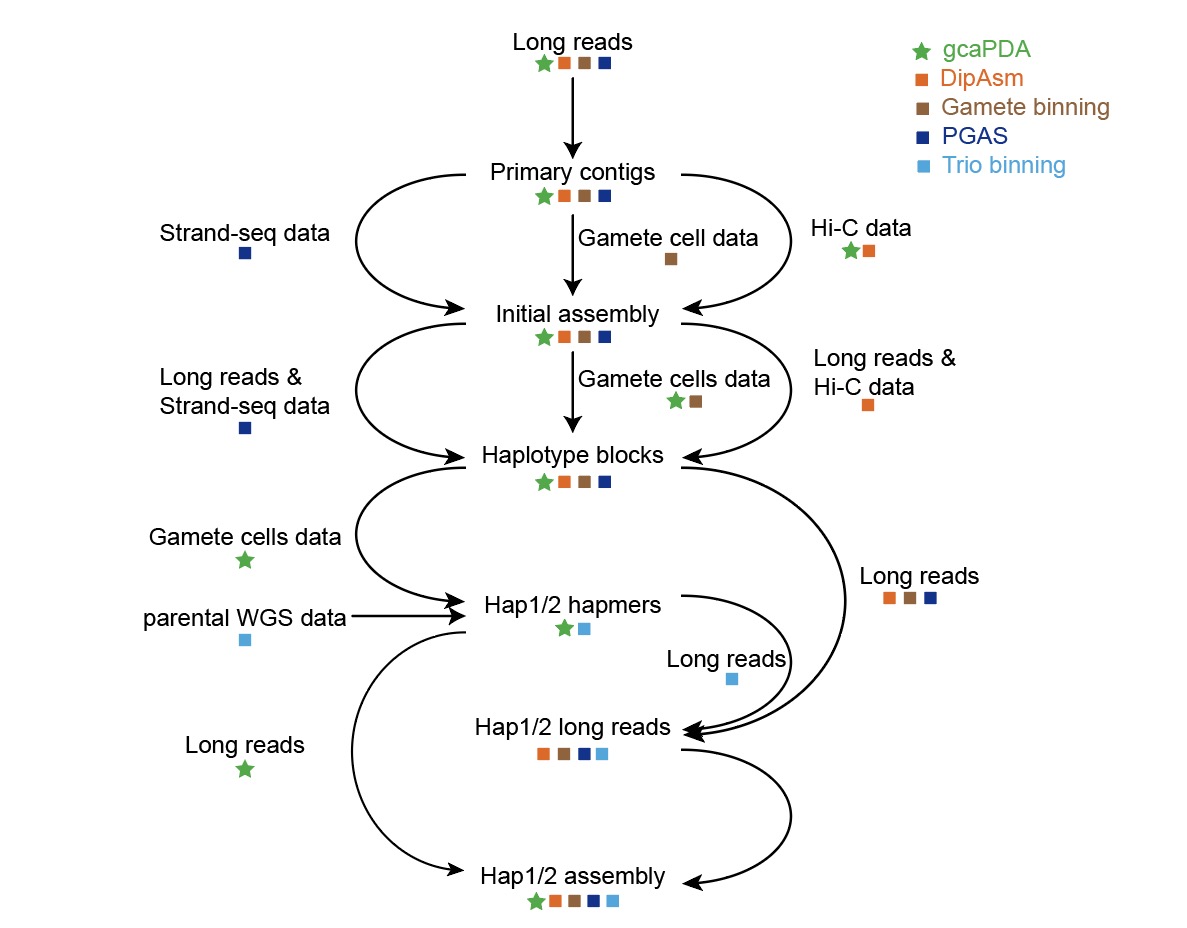


**Supplementary Figure 1.** **Comparison of framework of different diploid assemblers.** DipAsm, PGAS, Gamete-binning and gcaPDA starts with assembling long reads into primary contigs and scaffolding contigs into superscaffolds (initial assembly). Reads data was then mapped to the initial assembly to identify and phase variations into haplotype blocks. In DipAsm, gamete binning and PGAS, long reads were partitioned into haplotypes based on variations and each haplotype was assembled from partitioned long reads, respectively. In trio binning method, long reads were partitioned into haplotypes based on hapmers derived from parental WGS data and each haplotype was assembled from partitioned long reads, respectively. In contrast, gcaPDA partitioned gamete cell reads based on haplotype blocks to and generated hapmers. In gcaPDA, unpartitioned long reads and hapmers were used to assemble both haplotypes simultaneously. Data and analysis steps used in gcaPDA were indicated by green stars, while data and analysis steps used in DipAsm, gamete binning, PGAS and trio binning were indicated by orange, brown, deep blue and light blue cubes, respectively.


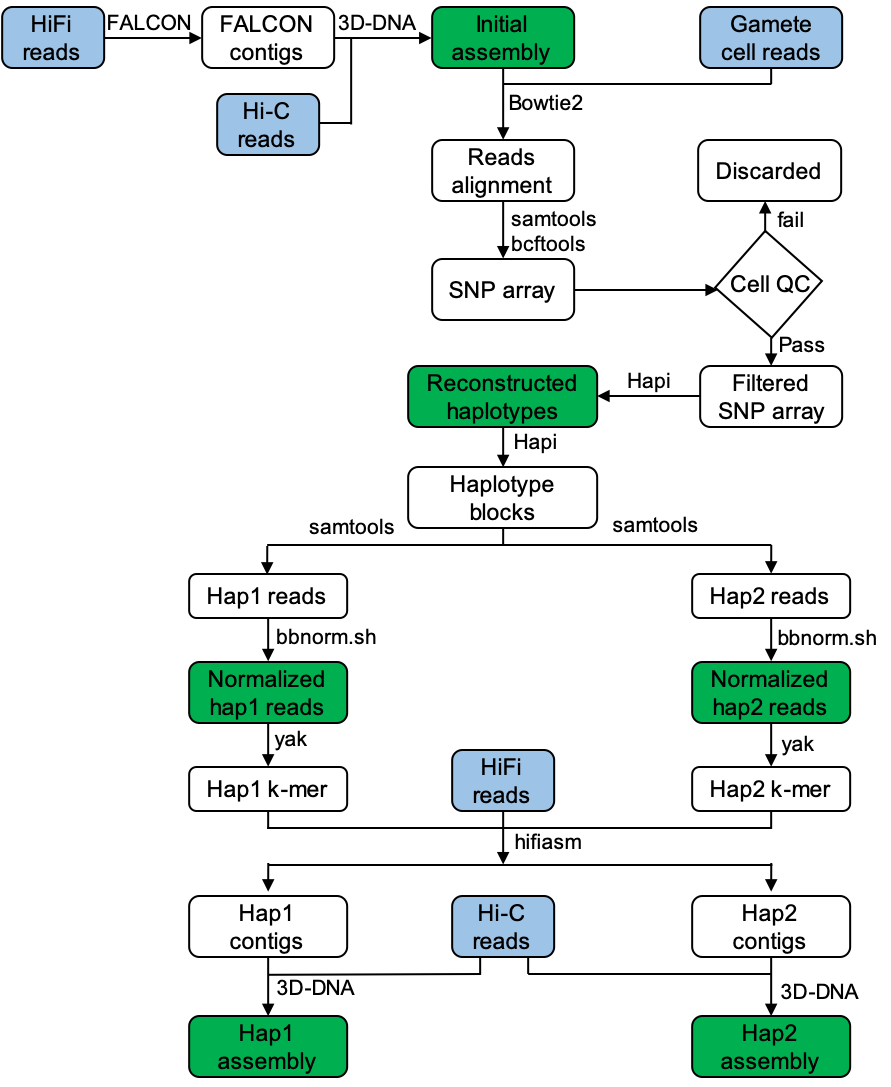


**Supplementary Figure 2**. **The analysis workflow of gcaPDA.** gcaPDA consists of 4 major steps: 1) building an initial assembly; 2) reconstruction of haplotypes; 3) partition and normalization of gamete cell reads and 4) generating chromosome-scale phased diploid assembly. Data are shown in round rectangles. Input data set (HiFi reads, Hi-C reads, gamete cell reads) highlighted in blue and result of each major step is highlighted in green. Software used at each analysis step is shown on the top/right.


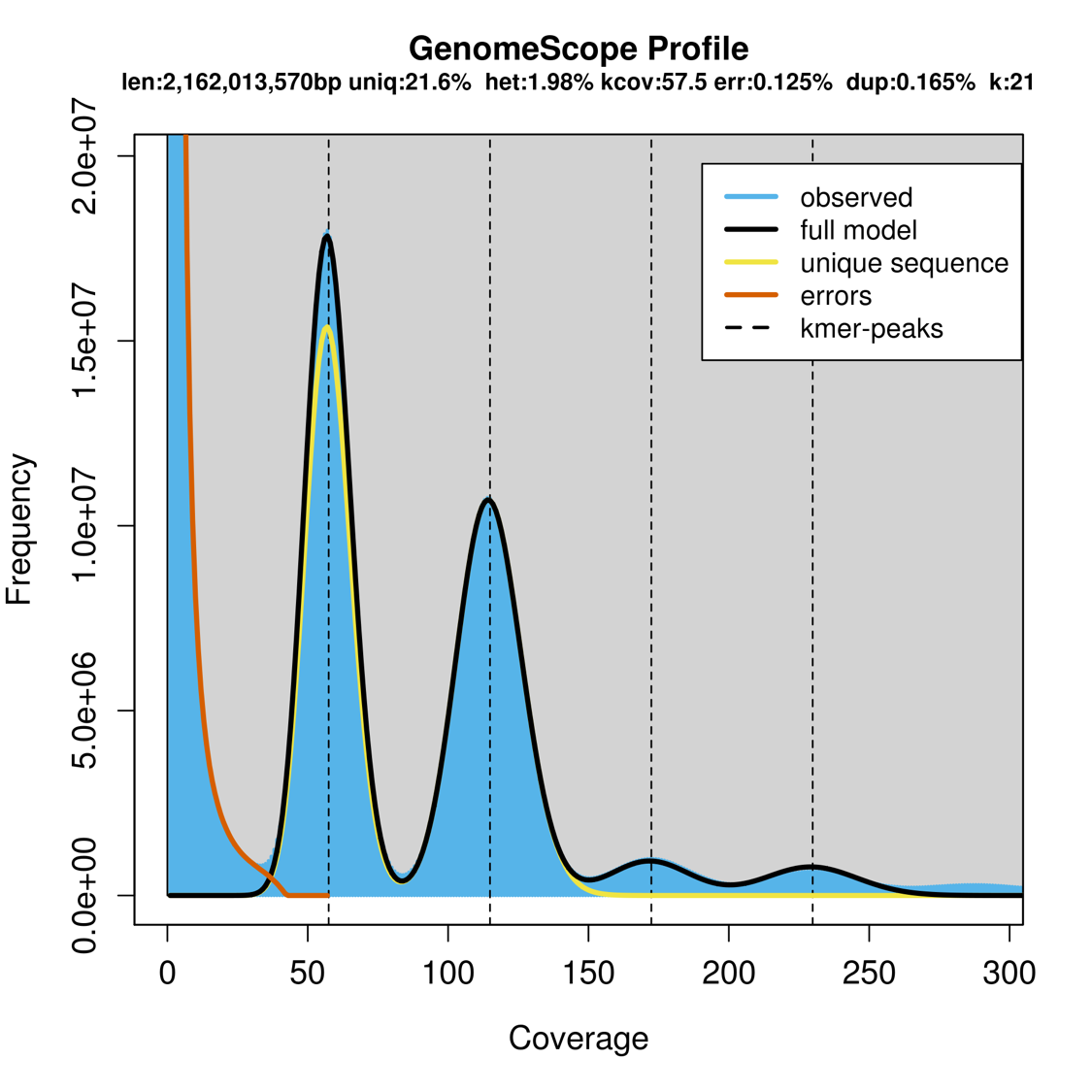


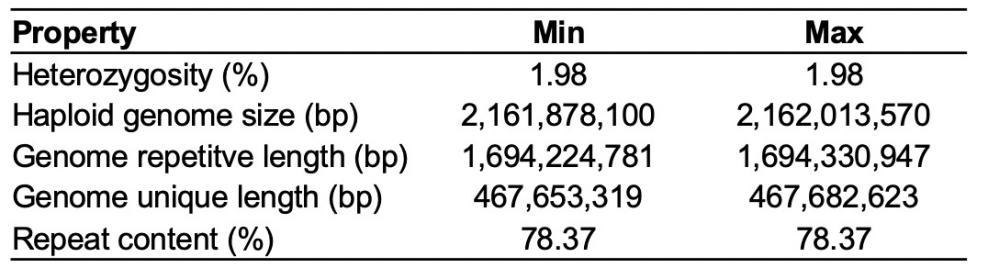


**Supplementary Figure 3.** **Genome survey of the SK x B73 F_1_ hybrid.** Simulated HiFi reads were used for this analysis (k-mer size =21). Haploid genome size and heterozygosity of F_1_ hybrid was estimated as 2.16 Gb and 1.98%, respectively.


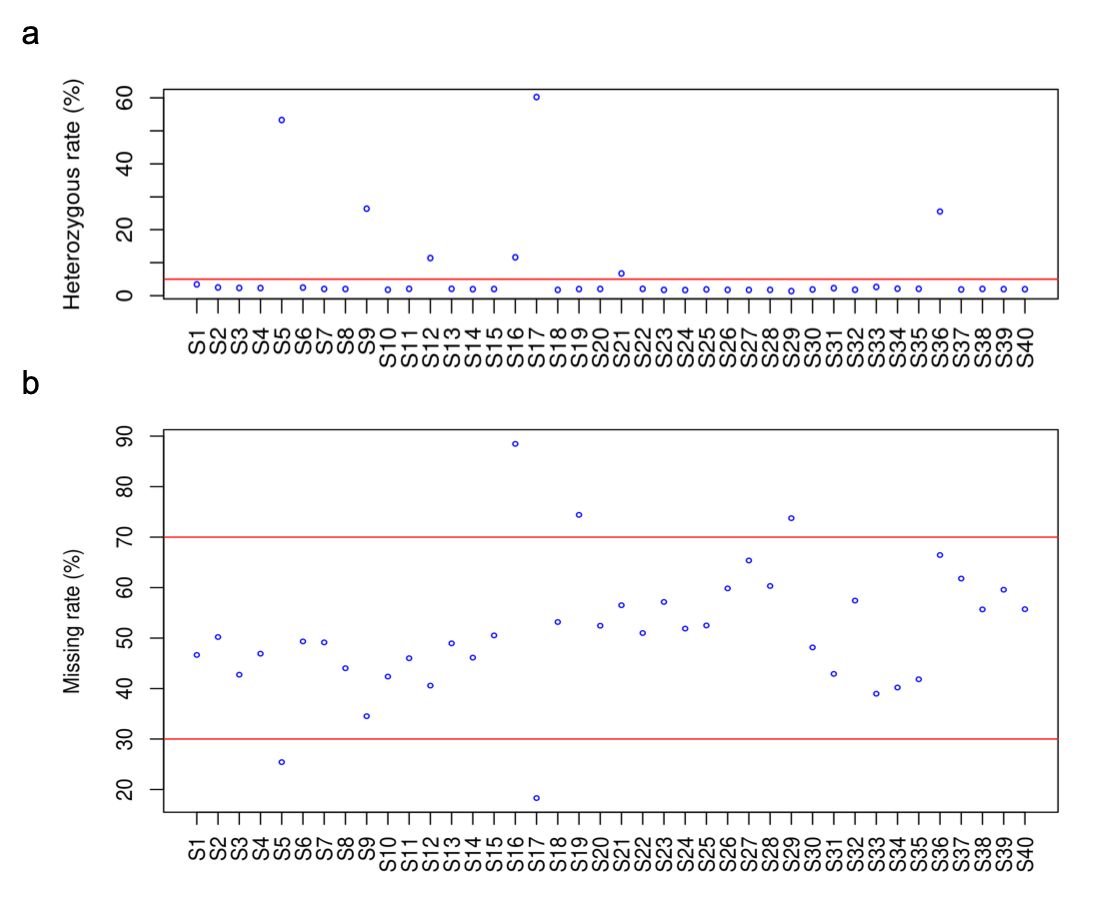


**Supplementary Figure 4**. **Quality control of gamete cells based on SNP statistics**. **a**), heterozygous rate of SNPs of gamete cells. Gamete cell with heterozygous rate higher that 5% (cutoff indicated by red line) were considered as contaminated and excluded from downstream analysis. **b**), SNP missing rate of gamete cells. Gamete cells with SNP missing rate >70% or <30% were considered as low-quality cells and excluded from downstream analysis.


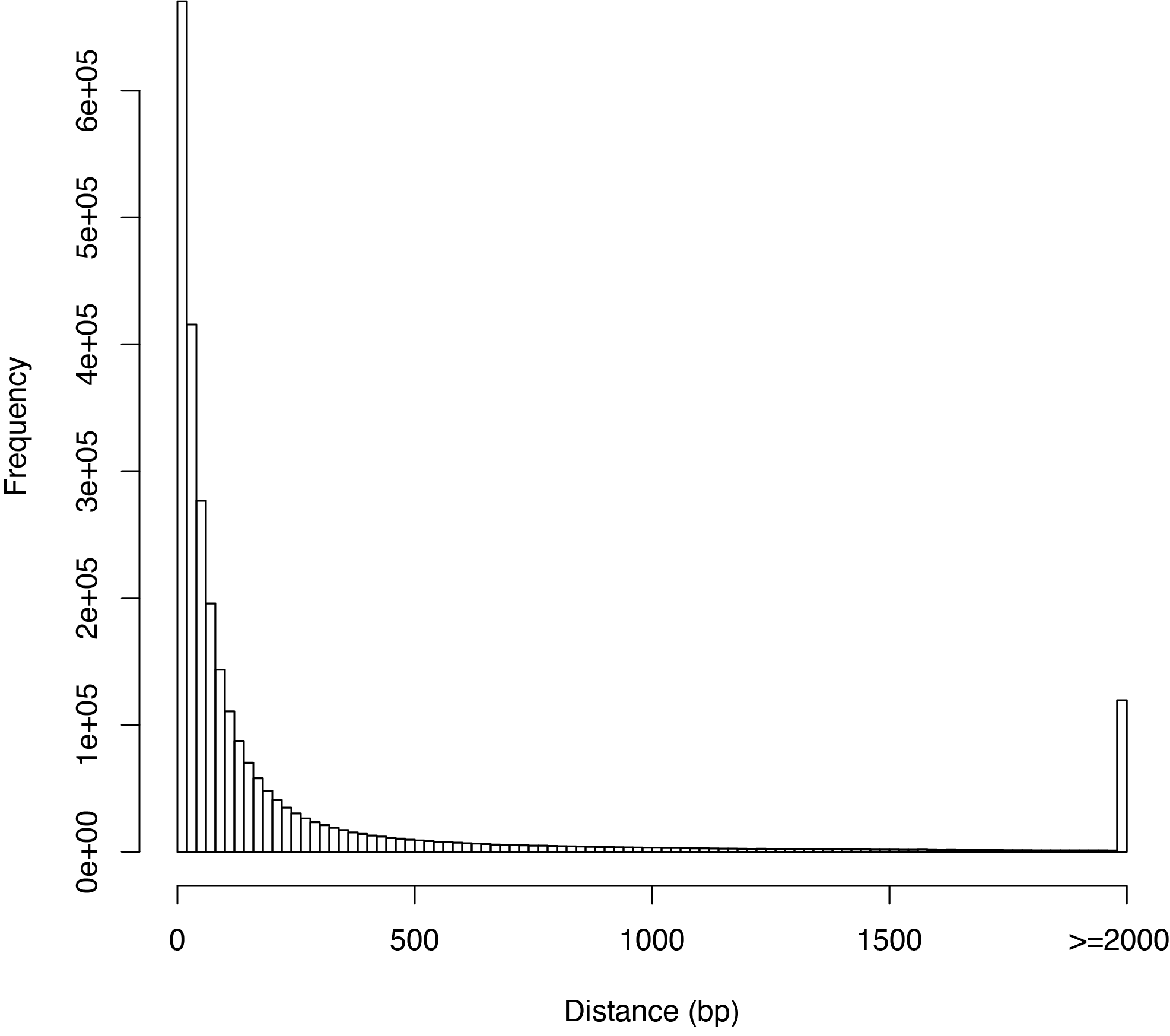


**Supplementary Figure 5.** **Distribution of distance between adjacent SNPs in the reconstructed haplotypes.**


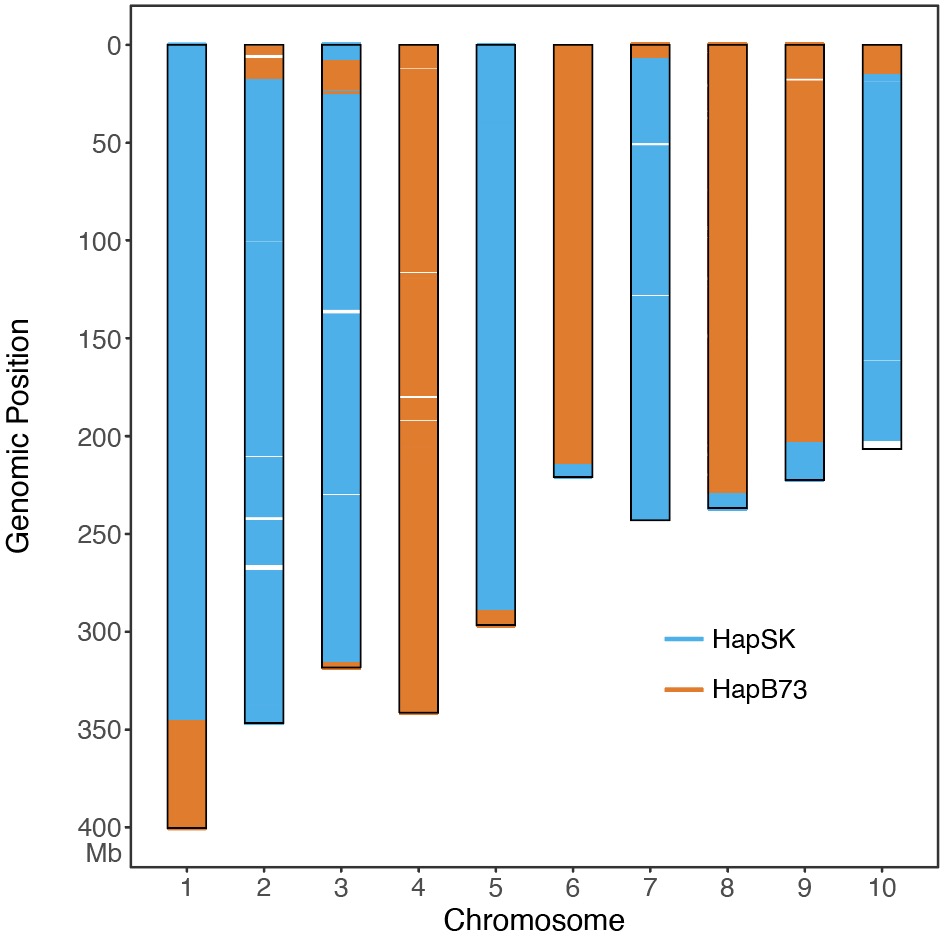


**Supplementary Figure 6.** **Visualization of haplotype blocks of gamete cell S2.** Chromosomes of the initial assembly was depicted with black rectangles. The genotype of gamete cell S2 at each SNP locus was compared with the reconstructed haplotype. SNP loci with identical genotype to hapSK were depicted with blue line, while SNP loci with identical genotype to hapB73 were depicted with orange line.


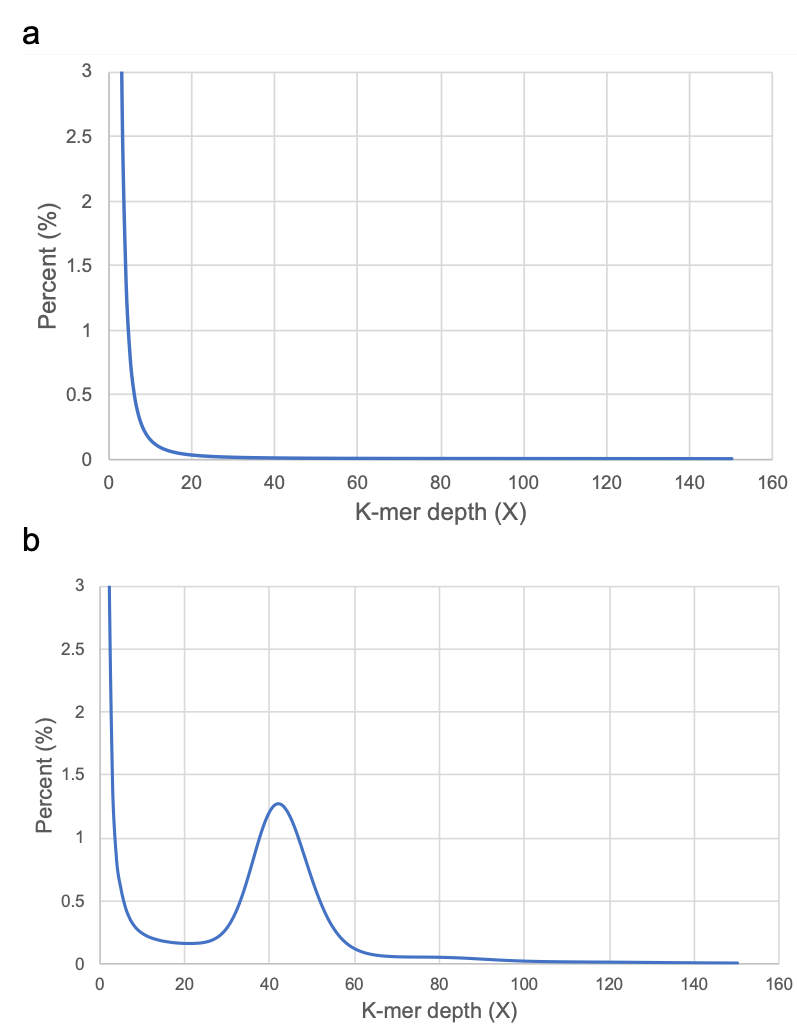


**Supplementary Figure 7**. K-mer distribution of haplotype reads before **a**) and after normalization **b**). We used k-mer size of length 31 for this analysis.


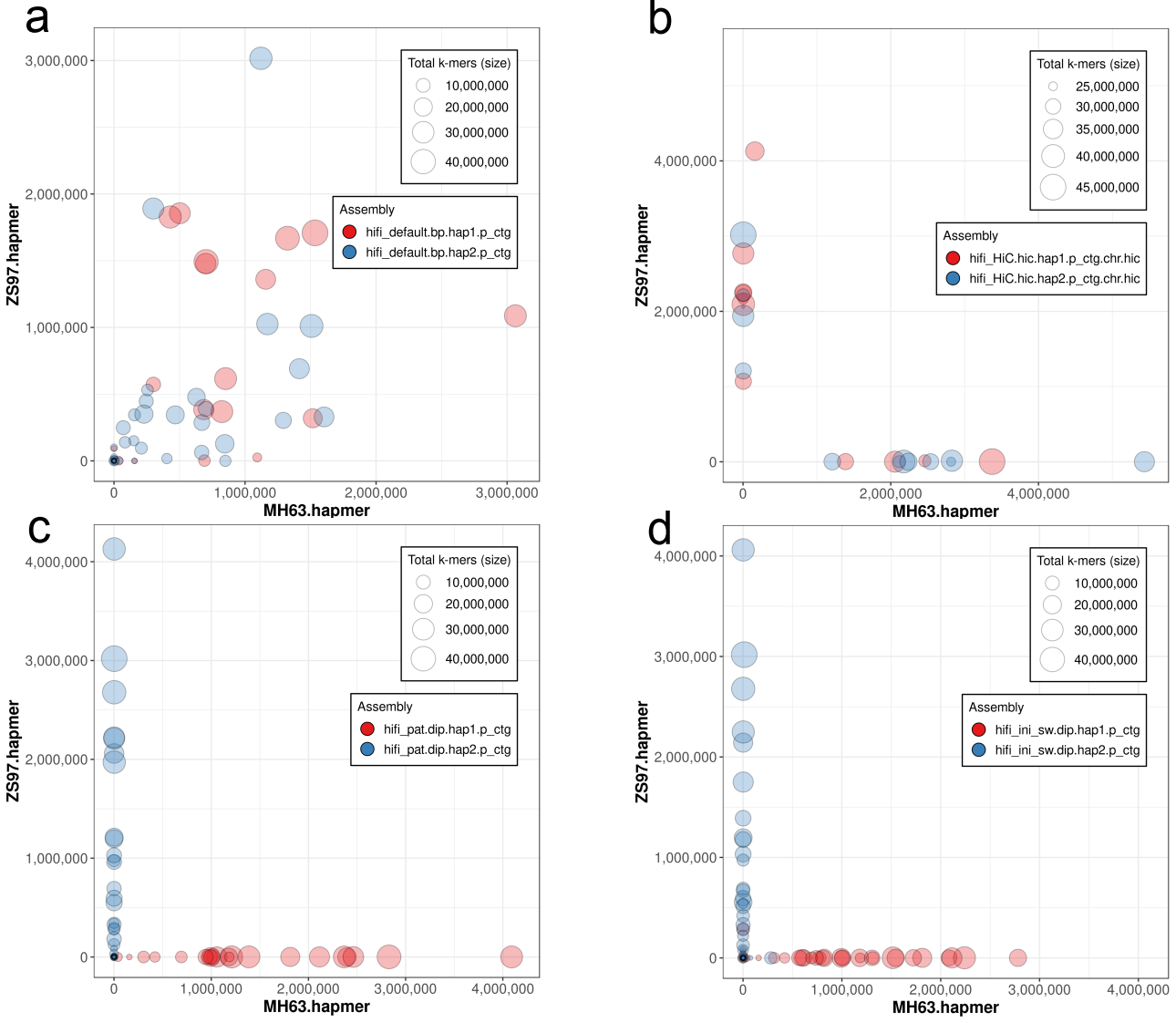


**Supplementary Figure 8.** Comparison of phasing accuracy of contigs of different assemblies for rice. Each contig is represented by a circle, with circles of primary/hapMH63 contigs filled red and alternative/hapZS97 contigs filled blue. The size of a circle is proportional to the total number of k-mer in the contig. The x and y axes refer to the number of MH63 hapmer and ZS97 hapmer identified in a contig, respectively. Panel **a)** Hifiasm assembly, **b)** Hifiasm+Hi-C assembly, **c)** Trio assembly, **d)** gcaPDA assembly.


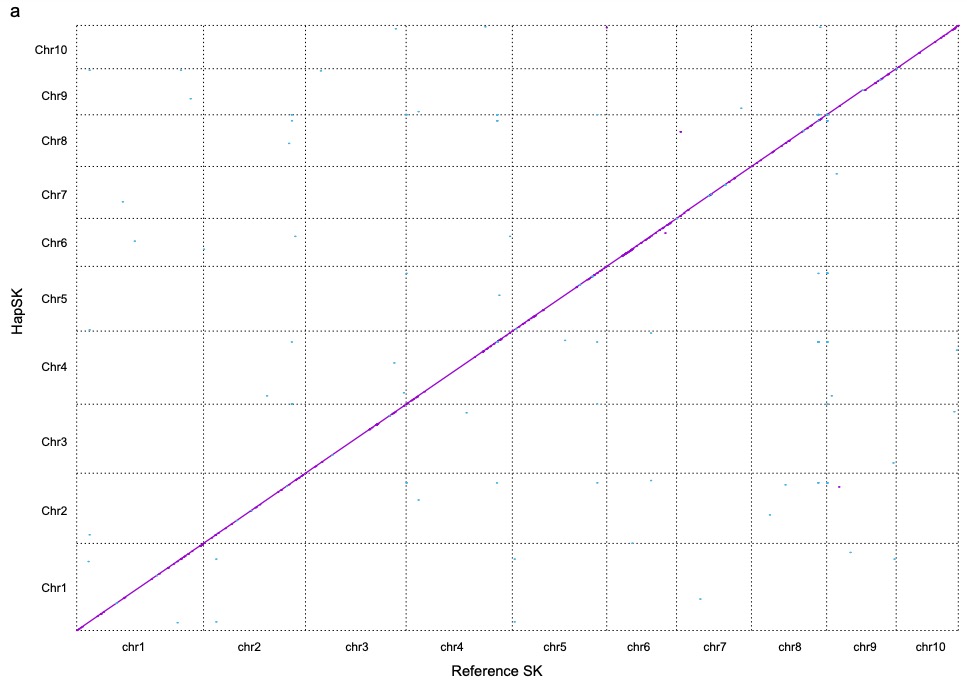


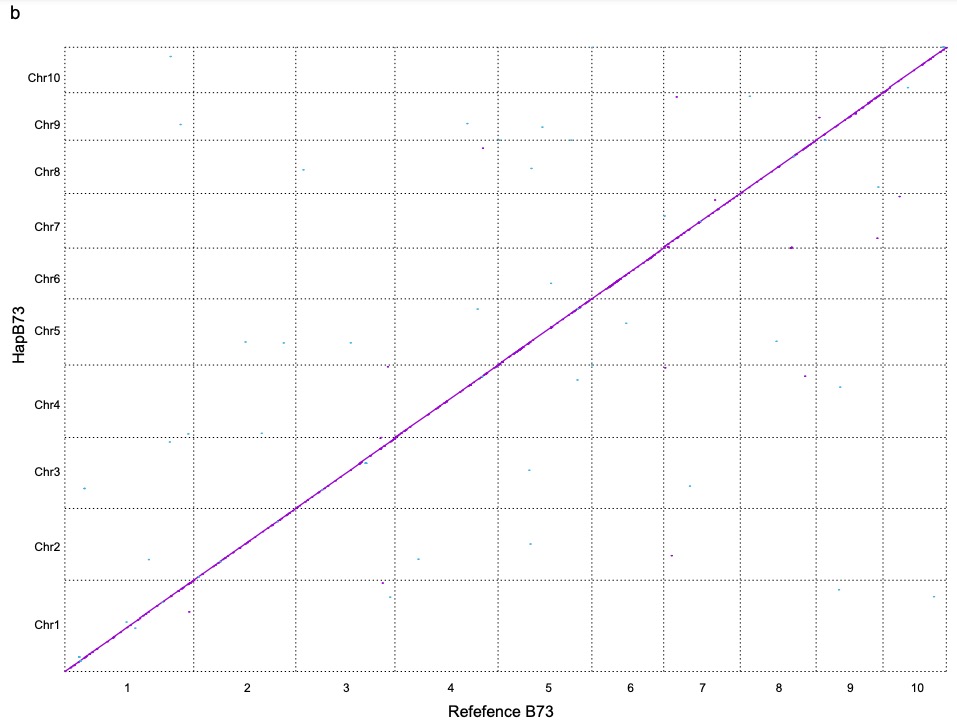


**Supplementary Figure 9.** Visualization of sequence alignments between gcaDPA assembly and parental genomes. a) Visualization of alignments between hapSK chromosomes and reference SK chromosomes. b) Visualization of alignments between hapB73 chromosomes and reference B73 chromosomes.

**Supplementary Figure 10.** Accumulated percentage of genomic k-mers covered in gamete cells reads. Genomic k-mers set were calculated from SK, B73 genome, or both (SK+B73). A genomic k-mer was defined was covered if its frequency in gamete cell reads is >4. A simulated accumulation curve was also plotted, assuming that each gamete cell randomly covered 50% of genomic k-mers.

**Supplementary Tables**

**Supplementary Table 1.** Statistics of simulated reads.

| **Simulated data set** | **Error rate** | **Read No.** | **Total read length (bp)** | **Average read length (bp)** |
| --- | --- | --- | --- | --- |
| B73 HiFi reads | 0.002 | 9,042,308 | 126,261,011,397 | 13,963 |
| SK HiFi reads | 0.002 | 9,255,246 | 129,233,929,145 | 13,963 |
| B73 short reads | 0.01 | 600,000,000 | 90,000,000,000 | 150 |
| SK short reads | 0.01 | 600,000,000 | 90,000,000,000 | 150 |

**Supplementary Table 2.** Statistics of sequencing reads of gamete cells.

| Sample | Raw reads | | Clean reads | |
| --- | --- | --- | --- | --- |
|  | Count (million) | Read base (Gb) | Count (million) | Read base (Gb) |
| S1 | 399.1 | 59.9 | 375.1 | 55.2 |
| S2 | 415.2 | 62.3 | 389.8 | 57.4 |
| S3 | 417.6 | 62.6 | 401.8 | 59.4 |
| S4 | 400.1 | 60.0 | 384.6 | 56.8 |
| S5 | 393.3 | 59.0 | 379.4 | 55.9 |
| S6 | 415.1 | 62.3 | 401.5 | 59.2 |
| S7 | 500.7 | 75.1 | 488.9 | 72.5 |
| S8 | 494.2 | 74.1 | 482.8 | 71.6 |
| S9 | 523.0 | 78.5 | 510.8 | 75.7 |
| S10 | 523.9 | 78.6 | 512.4 | 76.0 |
| S11 | 493.3 | 74.0 | 471.6 | 69.6 |
| S12 | 518.8 | 77.8 | 497.9 | 73.5 |
| S13 | 369.4 | 55.4 | 344.2 | 50.5 |
| S14 | 380.7 | 57.1 | 355.4 | 52.2 |
| S15 | 345.3 | 51.8 | 321.3 | 47.2 |
| S16 | 338.0 | 50.7 | 317.1 | 46.6 |
| S17 | 508.9 | 76.3 | 492.2 | 72.7 |
| S18 | 501.0 | 75.2 | 487.3 | 72.1 |
| S19 | 457.6 | 68.6 | 443.2 | 65.5 |
| S20 | 483.9 | 72.6 | 469.1 | 69.3 |
| S21 | 437.0 | 65.5 | 421.3 | 62.1 |
| S22 | 382.9 | 57.4 | 368.3 | 54.3 |
| S23 | 376.6 | 56.5 | 364.2 | 53.7 |
| S24 | 443.0 | 66.5 | 429.7 | 63.5 |
| S25 | 394.1 | 59.1 | 379.6 | 56.1 |
| S26 | 326.3 | 48.9 | 314.0 | 46.4 |
| S27 | 482.0 | 72.3 | 468.0 | 69.3 |
| S28 | 371.4 | 55.7 | 359.8 | 53.3 |
| S29 | 429.3 | 64.4 | 415.6 | 61.5 |
| S30 | 413.6 | 62.0 | 398.6 | 58.8 |
| S31 | 413.8 | 62.1 | 400.5 | 59.2 |
| S32 | 463.6 | 69.5 | 449.3 | 66.5 |
| S33 | 289.0 | 43.4 | 270.9 | 39.6 |
| S34 | 366.5 | 55.0 | 346.7 | 50.7 |
| S35 | 307.9 | 46.2 | 289.1 | 42.3 |
| S36 | 337.8 | 50.7 | 319.1 | 46.7 |
| S37 | 404.7 | 60.7 | 392.1 | 58.0 |
| S38 | 463.8 | 69.6 | 448.8 | 66.3 |
| S39 | 393.2 | 59.0 | 379.1 | 56.0 |
| S40 | 455.8 | 68.4 | 438.4 | 64.7 |

**Supplementary Table 3**. Statistics of Hi-C sequencing reads.

| **Tissue** | **Total read pair** | **Total read length (bp)** |
| --- | --- | --- |
| Roots | 1,423,190,273 | 426,957,081,900 |

**Supplementary Table 4**. Purge haplotigs from FALCON primary contigs.

| **Contigs** | **Primary contigs** | **Artefacts** | **Haplotigs** | **Clean contigs** |
| --- | --- | --- | --- | --- |
|  |  |  |  |  |
| Contig No. | 5,014 | 141 | 2,462 | 2,411 |
| Contig N50 (bp) | 1,707,428 | 24,630 | 357,307 | 2,017,689 |
| Contig length (bp) | 2,903,282,308 | 2,896,924 | 428,970,525 | 2,471,414,859 |

**Supplementary Table 5**. Statistics of SNPs identified in gamete cells.

| Sample | Homozygous calls | Heterozygous calls | Missing calls | Heterozygous rate (%) | Missing rate (%) |
| --- | --- | --- | --- | --- | --- |
| S1 | 2,100,669 | 74,653 | 1,902,968 | 3.43 | 46.66 |
| S2 | 1,980,218 | 50,490 | 2,047,582 | 2.49 | 50.21 |
| S3 | 2,280,119 | 54,649 | 1,743,522 | 2.34 | 42.75 |
| S4 | 2,115,073 | 49,978 | 1,913,239 | 2.31 | 46.91 |
| S5^*^ | 1,421,017 | 1,620,736 | 1,036,537 | 53.28 | 25.42 |
| S6 | 2,015,379 | 50,726 | 2,012,185 | 2.46 | 49.34 |
| S7 | 2,031,329 | 42,354 | 2,004,607 | 2.04 | 49.15 |
| S8 | 2,236,783 | 46,034 | 1,795,473 | 2.02 | 44.03 |
| S9^*^ | 1,964,351 | 705,800 | 1,408,139 | 26.43 | 34.53 |
| S10 | 2,307,611 | 42,321 | 1,728,358 | 1.80 | 42.38 |
| S11 | 2,155,980 | 45,819 | 1,876,491 | 2.08 | 46.01 |
| S12^*^ | 2,146,651 | 276,147 | 1,655,492 | 11.40 | 40.59 |
| S13 | 2,038,436 | 43,255 | 1,996,599 | 2.08 | 48.96 |
| S14 | 2,153,800 | 43,283 | 1,881,207 | 1.97 | 46.13 |
| S15 | 1,977,077 | 40,646 | 2,060,567 | 2.01 | 50.53 |
| S16^*^ | 415,674 | 54,632 | 3,607,984 | 11.62 | 88.47 |
| S17^*^ | 1,324,847 | 2,007,100 | 746,343 | 60.24 | 18.30 |
| S18 | 1,875,931 | 33,704 | 2,168,655 | 1.76 | 53.18 |
| S19^*^ | 1,022,559 | 20,965 | 3,034,766 | 2.01 | 74.41 |
| S20 | 1,899,502 | 39,603 | 2,139,185 | 2.04 | 52.45 |
| S21^*^ | 1,654,560 | 119,133 | 2,304,597 | 6.72 | 56.51 |
| S22 | 1,957,133 | 41,323 | 2,079,834 | 2.07 | 51.00 |
| S23 | 1,716,481 | 30,664 | 2,331,145 | 1.76 | 57.16 |
| S24 | 1,928,832 | 33,566 | 2,115,892 | 1.71 | 51.88 |
| S25 | 1,900,530 | 36,837 | 2,140,923 | 1.90 | 52.50 |
| S26 | 1,608,829 | 29,059 | 2,440,402 | 1.77 | 59.84 |
| S27 | 1,387,594 | 24,601 | 2,666,095 | 1.74 | 65.37 |
| S28 | 1,589,609 | 28,721 | 2,459,960 | 1.77 | 60.32 |
| S29^*^ | 1,055,719 | 14,869 | 3,007,702 | 1.39 | 73.75 |
| S30 | 2,074,821 | 39,686 | 1,963,783 | 1.88 | 48.15 |
| S31 | 2,275,225 | 53,102 | 1,749,963 | 2.28 | 42.91 |
| S32 | 1,704,902 | 31,292 | 2,342,096 | 1.80 | 57.43 |
| S33 | 2,423,031 | 65,624 | 1,589,635 | 2.64 | 38.98 |
| S34 | 2,386,849 | 51,447 | 1,639,994 | 2.11 | 40.21 |
| S35 | 2,322,732 | 49,391 | 1,706,167 | 2.08 | 41.84 |
| S36^*^ | 1,018,799 | 349,340 | 2,710,151 | 25.53 | 66.45 |
| S37 | 1,529,145 | 29,355 | 2,519,790 | 1.88 | 61.79 |
| S38 | 1,770,185 | 37,122 | 2,270,983 | 2.05 | 55.68 |
| S39 | 1,615,903 | 32,695 | 2,429,692 | 1.98 | 59.58 |
| S40 | 1,770,442 | 35,391 | 2,272,457 | 1.96 | 55.72 |

^*^The nine gamete cells that failed quality control were marked with asterisk.

**Supplementary Table 6.** The statistic of HiFi reads of the MH63×ZS97 hybrid.

| **Read type** | **Read count** | **Read length N50 (bp)** | **Average read length (bp)** | **Total data (bp)** |
| --- | --- | --- | --- | --- |
| HiFi | 1,576,247 | 15,156 | 14,937 | 23,544,544,010 |

**Supplementary Table 7.** The statistic of single cell sequencing data of the tetrads of MH63×ZS97 hybrid**.**

| Sample | Raw reads | | Clean reads | |
| --- | --- | --- | --- | --- |
|  | Read count | Base | Read count | Base |
| RT41 | 58,254,492 | 8,759,015,552 | 56,287,660 | 8,265,514,514 |
| RT42 | 83,846,040 | 12,609,767,193 | 81,268,224 | 11,945,643,467 |
| RT43 | 83,406,624 | 12,542,300,431 | 80,926,474 | 11,901,761,332 |
| RT44 | 100,654,960 | 15,135,314,658 | 97,689,520 | 14,370,727,281 |
| RT71 | 72,971,540 | 10,977,960,671 | 70,229,432 | 10,272,436,005 |
| RT72 | 72,802,936 | 10,953,003,560 | 70,403,962 | 10,314,726,281 |
| RT73 | 63,509,170 | 9,555,855,721 | 60,300,104 | 8,778,729,731 |
| RT74 | 64,248,898 | 9,666,357,008 | 61,895,344 | 9,060,409,870 |
| SY11-1 | 109,967,772 | 16,495,165,800 | 109,432,534 | 16,225,424,636 |
| SY11-2 | 86,458,770 | 12,968,815,500 | 85,939,628 | 12,725,411,365 |
| SY11-3 | 82,510,882 | 12,376,632,300 | 82,121,684 | 12,167,692,258 |
| SY11-4 | 39,351,486 | 5,902,722,900 | 38,687,906 | 5,657,186,898 |
| SY4-1 | 72,181,524 | 10,827,228,600 | 71,801,426 | 10,638,655,087 |
| SY4-2 | 70,223,826 | 10,533,573,900 | 69,889,316 | 10,366,621,819 |
| SY4-3 | 75,152,336 | 11,272,850,400 | 74,723,426 | 11,048,999,123 |
| SY4-4 | 66,906,102 | 10,035,915,300 | 66,528,532 | 9,853,820,997 |
| SY6-1 | 59,872,644 | 8,980,896,600 | 59,007,468 | 8,641,903,301 |
| SY6-2 | 58,286,934 | 8,743,040,100 | 57,509,056 | 8,435,277,259 |
| SY6-3 | 57,722,378 | 8,658,356,700 | 57,005,230 | 8,368,916,750 |
| SY6-4 | 85,273,448 | 12,791,017,200 | 84,831,298 | 12,572,792,007 |
| SY9-1 | 53,626,392 | 8,043,958,800 | 53,280,868 | 7,837,375,791 |
| SY9-2 | 92,419,054 | 13,862,858,100 | 91,774,086 | 13,561,021,661 |
| SY9-3 | 77,274,726 | 11,591,208,900 | 76,593,756 | 11,297,219,604 |
| SY9-4 | 76,722,502 | 11,508,375,300 | 76,273,632 | 11,270,407,794 |

**Supplementary Table 8.** The statistic of Hi-C reads of the MH63×ZS97 hybrid.

| **Tissue** | **Total read pair** | **Total read length (bp)** |
| --- | --- | --- |
| Root of rice F1 | 677,267,436 | 101,590,115,400 |

**Supplementary Table 9.** Evaluation of Hifiasm assembly, Trio assembly and gcaPDA assembly.

| **Assembly** | **Contigs** | | | **Kmer completeness (%)** | | | | **Gene completeness (%)** | | | |
| --- | --- | --- | --- | --- | --- | --- | --- | --- | --- | --- | --- |
|  | **Total (Mb)** | **# Number** | **N50 (Mb)** | **All** | **Hap MH63** | **Hap ZS97** | **PPV** | **Comp** | **Dup.** | **Frag.** | **Mis.** |
| **Gapless reference** |  |  |  |  |  |  |  |  |  |  |  |
| **MH63 genome** | 396 | 12 | 31.9 | 89.07 | 99.86 | 0.03 | 99.98 | 98.4 | 0.6 | 0.2 | 1.4 |
| **ZS97 genome** | 392 | 12 | 32.1 | 88.61 | 0.04 | 99.92 | 99.96 | 98.4 | 0.6 | 0.2 | 1.4 |
| **MH63 + ZS97 genome** | 787 | 24 | 32.1 | 99.23 | 99.86 | 99.92 | 99.97 | 98.4 | 98.3 | 0.2 | 1.4 |
| **Hifiasm assembly** |  |  |  |  |  |  |  |  |  |  |  |
| Hap1 contigs | 413 | 254 | 31.6 | 89.44 | 50.54 | 54.62 | 50.66 | 98.4 | 0.6 | 0.2 | 1.4 |
| Hap2 contigs | 390 | 108 | 21.2 | 87.47 | 50.50 | 46.26 | 45.22 | 97.6 | 0.6 | 0.3 | 2.1 |
| Hap1+Hap2 contigs | 803 | 362 | 28.3 | 99.31 | 99.97 | 99.94 | 48.05 | 98.8 | 97.3 | 0.2 | 1.0 |
| **Trio-bin assembly** |  |  |  |  |  |  |  |  |  |  |  |
| MH63 contigs | 409 | 253 | 25.6 | 89.18 | 99.98 | 0.06 | 99.95 | 98.5 | 0.6 | 0.2 | 1.3 |
| ZS97 contigs | 396 | 137 | 27.9 | 87.69 | 0.07 | 99.98 | 99.93 | 97.3 | 0.6 | 0.2 | 2.5 |
| MH63 + ZS97 contigs | 804 | 390 | 25.6 | 99.31 | 99.98 | 99.98 | 99.94 | 98.8 | 97.0 | 0.2 | 1.0 |
| **gcaPDA assembly** |  |  |  |  |  |  |  |  |  |  |  |
| hapMH63 contigs | 408 | 303 | 15.7 | 88.87 | 99.01 | 1.69 | 98.48 | 98.2 | 0.6 | 0.3 | 1.5 |
| HapZS97 contigs | 397 | 125 | 19.1 | 87.97 | 1.35 | 98.66 | 98.51 | 97.6 | 0.6 | 0.3 | 2.1 |
| HapMH63 +hapZS97 assembly | 805 | 428 | 17.6 | 99.31 | 99.97 | 99.97 | 98.50 | 98.6 | 97.2 | 0.3 | 1.1 |

**Supplementary Table 10.** Statistics of haplotype blocks from the other haplotype.

|  | **Chromosomes** | | **Un-anchored sequences** | |
| --- | --- | --- | --- | --- |
|  | **Length (bp)** | **Percent (%)** | **Length (bp)** | **Percent (%)** |
| HapSK assembly | 2,086,840,877 | 96.52 | 75,287,149 | 3.48 |
| HapB73 assenbly | 2,104,568,114 | 97.48 | 54,428,728 | 2.52 |
| HapB73 blocks in HapSK assembly | 22,445,582 | 1.08 | 22,468,175 | 29.84 |
| HapSK blocks in HapB73 assembly | 31,273,932 | 1.49 | 42,870,239 | 78.76 |
| Total mis-assigned blocks | 53,719,514 | 1.28 | 65,338,414 | 50.37 |

**Supplementary Table 11.** Statistics of whole genome sequence comparison between haplotype assemblies and reference assemblies.

| **Comparison** | **HapSK vs SK reference** | | **HapB73 vs B73 reference** | |
| --- | --- | --- | --- | --- |
|  | **HapSK** | **SK reference** | **HapB73** | **B73 reference** |
| Total bases (bp) | 2,162,128,026 | 2,153,898,797 | 2,158,996,842 | 2,104,350,182 |
| Alignment coverage (%) | 99.03 | 99.04 | 98.05 | 99.72 |
| 1-1 alignment identity (%) | 99.99 | 99.99 | 99.99 | 99.99 |
| Relocations | 28 | 28 | 20 | 45 |
| Translocations | 81 | 47 | 167 | 50 |
| Inversions | 6 | 5 | 3 | 5 |
| Insertions (>1bp) | 1,822 | 2,051 | 2,848 | 963 |
| SNPs | 17,132 | 17,132 | 19,830 | 19,830 |
| Indels (1bp) | 22,560 | 22,560 | 36,695 | 36,695 |

**Supplementary Table 12.** The computing resources used by differnet methods for rice F_1_.

|  |  | CPU hours | | | |
| --- | --- | --- | --- | --- | --- |
|  |  | gcaPDA | hifiasm | hifiasm+hic | hifiasm + parental WGS |
| Initial assembly | FALCON | 3,600.00 |  |  |  |
| hi-c scaffolding | 3d-dna pipeline | 201.00 |  |  |  |
| Mapping | gamete cell Read QC | 5.57 |  |  |  |
|  | bowtie index | 0.45 |  |  |  |
|  | bowtie mapping | 268.96 |  |  |  |
|  | sam to bam | 10.65 |  |  |  |
| SNP calling | bcftools mpileup | 59.68 |  |  |  |
|  | bcftools call variant | 0.54 |  |  |  |
| Extracting haplotypic reads | Hapi.R | 1.27 |  |  |  |
|  | Extract hap bams | 11.10 |  |  |  |
|  | merge hap bam | 51.34 |  |  |  |
| Coverage normalization | sort hap bam |  |  |  |  |
|  | bam to fastq |  |  |  |  |
|  | bbnorm | 176.59 |  |  |  |
| de novo assembly | yak | 7.25 |  |  | 6.65 |
|  | hifiasm v20211003 | 163.20 | 156.95 | 160.56 | 149.86 |
|  | **Total** | **4,557.61** | **156.95** | **160.56** | **156.51** |

**Supplementary Table 13.** The computing resources used by differnet methods for maize F_1_.

|  |  | CPU hours | | | |
| --- | --- | --- | --- | --- | --- |
|  |  | gcaPDA | hifiasm | hifiasm+hic | hifiasm + parental WGS |
| Initial assembly | FALCON | 15,000.00 |  |  |  |
| hi-c scaffolding | 3d-dna pipeline | 3,449.13 |  |  |  |
| Mapping | Read QC | 389.51 |  |  |  |
|  | bowtie index | 3.00 |  |  |  |
|  | bowtie mapping | 6,000.00 |  |  |  |
|  | sam to bam | 160.00 |  |  |  |
| SNP calling | bcftools mpileup | 2,000.00 |  |  |  |
|  | bcftools call variant | 12.00 |  |  |  |
| Extracting haplotypic reads | Hapi.R | 35.00 |  |  |  |
|  | Extract hap bams | 713.00 |  |  |  |
|  | merge hap bam | 1,152.00 |  |  |  |
| Coverage normalization | sort hap bam |  |  |  |  |
|  | bam to fastq |  |  |  |  |
|  | bbnorm | 1,782.00 |  |  |  |
| de novo assembly | yak | 21.40 |  |  | 23.00 |
|  | hifiasm v20211003 | 3,597.00 | 3,558.00 | 3,673.00 | 3,603.00 |
|  | **Total** | **34,314.04** | **3,558.00** | **3,673.00** | **3,626.00** |
